# Structure–Dopant Concentration Relations in Europium-Doped Yttrium Molybdate and Peak-Sharpening for Luminescence Temperature Sensing

**DOI:** 10.3390/ma17174267

**Published:** 2024-08-28

**Authors:** Tamara Gavrilović, Aleksandar Ćirić, Mina Medić, Zoran Ristić, Jovana Periša, Željka Antić, Miroslav D. Dramićanin

**Affiliations:** Center of Excellence for Photoconversion, Vinča Institute of Nuclear Sciences-National Institute of the Republic of Serbia, University of Belgrade, 11001 Belgrade, Serbia

**Keywords:** phosphor, luminescence thermometry, molybdates, europium

## Abstract

A set of Eu^3+^-doped molybdates, Y_2−_xEuxMo_3_O_12_ (x = 0.04; 0.16; 0.2; 0.4; 0.8; 1; 1.6; 2), was synthesized using a solid-state technique and their properties studied as a function of Eu^3+^ concentration. X-ray diffraction showed that the replacement of Y^3+^ with larger Eu^3+^ resulted in a transformation from orthorhombic (low doping concentrations) through tetragonal (high doping concentrations), reaching monoclinic structure for full replacement in Eu_2_Mo_3_O_12_. The intensity of typical Eu^3+^ red emission slightly increases in the orthorhombic structure then rises significantly with dopant concentration and has the highest value for the tetragonal Y_2_Mo_3_O_12_:80mol% Eu^3+^. Further, the complete substitution of Y^3+^ with Eu^3+^ in the case of monoclinic Eu_2_Mo_3_O_12_ leads to decreased emission intensity. Lifetime follows a similar trend; it is lower in the orthorhombic structure, reaching slightly higher values for the tetragonal structure and showing a strong decrease for monoclinic Eu_2_Mo_3_O_12_. Temperature-sensing properties of the sample with the highest red Eu^3+^ emission, Y_2_Mo_3_O_12_:80mol% Eu^3+^, were analyzed by the luminescence intensity ratio method. For the first time, the peak-sharpening algorithm was employed to separate overlapping peaks in luminescence thermometry, in contrast to the peak deconvolution method. The Sr (relative sensitivity) value of 2.8 % K^−1^ was obtained at room temperature.

## 1. Introduction

Single or composite materials that can be used for multiple applications are known as multifunctional materials. Those systems are “smart” because they react to external stimuli based on the material’s properties (mechanical, electrical, or optical). Multifunctional optical materials interact strongly with electromagnetic radiation in the spectrum’s visible, ultraviolet, and infrared regions. They can be used as the building blocks of innovative technologies with the potential to address different societal challenges. These materials have significant potential for increasing efficiency, safety, and responsiveness while reducing size, weight, cost, power consumption, and complexity in new system performance. Therefore, the identification of high-performance multifunctional optical materials is a continuous task. 

Herein, we propose Eu^3+^-doped molybdates as a promising luminescent material with potential applications (i) as a red phosphor in the field of lighting and displays and (ii) as a thermal probe in the luminescent thermometry field.

At present, red phosphors with a variety of luminescence centers are available in the field of lighting and displays but there is a space room for further improvement, in terms of:

(1) Ease of synthesis, cost-effectiveness, and long-term stability.
(2) Increased emission intensity and luminous efficacy for improved brightness and reduced energy consumption in WLED devices.
(3) Enhanced thermal stability of emission intensity to ensure that LEDs and displays function optimally at elevated temperatures.

Thus, advancing emission efficiency, fine-tuning luminescence spectra, and boosting thermal stability in red phosphors are crucial for the LED and display industries. 

Thermometry, the practice of measuring temperature, is currently performed using various devices based on different measurement principles. However, there is an ongoing demand for innovative measurement concepts and temperature probes, particularly for applications in emerging fields such as nanotechnology, biotechnology, and integrated optics. Today, there is an urgent need for non-contact thermometry for objects that are either in motion, sensitive to contact, hard to access, or located in hazardous environments. To meet these needs, temperature measurements that leverage changes in the optical properties of materials are seen as particularly promising, with a notable focus on the temperature-dependent luminescent properties of materials [1]. Like the field of lighting and displays, researchers reported numerous luminescence temperature probes; however, there is always room for improvement:

(1) Higher emission intensity for easy signal detection.
(2) Better sensitivity to temperature, namely higher relative sensitivity values.

In this research, Y_2_Mo_3_O_12_ was selected for the following reasons. Firstly, host materials with optically inactive rare-earth ions such as Y^3+^, La^3+^, Gd^3+^, and Lu^3+^ are suitable candidates for the incorporation of optically active lanthanides, to synthesize highly efficient luminescent materials. Secondly, these types of materials remain rather stable against environmental influence, are non-toxic, and have excellent chemical, thermal, and electrical stability [2,3]. Thirdly, molybdates form complex crystal structures with a set of temperature- and pressure-dependent phase transitions that show interesting luminescent properties [4,5,6,7,8,9,10,11,12]. In addition, when doped with Eu^3+^ ions Y_2_Mo_3_O_12_ shows efficient red photoluminescence necessary for the development of WLEDs with spectral characteristics comparable to daylight [13]. Also, rare-earth-doped molybdates have shown excellent performance in the field of luminescence thermometry due to the good thermal stability and realization of non-contact measurements with high precision and sensitivity [14,15,16,17,18,19,20,21].

In this paper, we studied the set of Eu^3+^-doped molybdates of general formula Y_2−x_Eu_x_Mo_3_O_12_ (x = 0.04; 0.16; 0.2; 0.4; 0.8; 1; 1.6 and 2). We showed how different Eu^3+^ dopant concentrations affect structural properties and induce phase transitions from orthorhombic via tetragonal to monoclinic. The optical properties of the set were investigated in detail using UV-VIS and photoluminescent spectroscopy. Further, luminescence thermometry using luminescence intensity ratio (LIR) in the 300 K to 650 K temperature range was demonstrated on the sample with the highest emission intensity. LIR is the most researched temperature-readout method in luminescence thermometry and among lanthanide-doped probes, LIR by Eu^3+^ shows the highest sensitivity. In Y_2_Mo_3_O_12_ Eu^3+^, the emissions of thermally coupled levels overlap. Traditionally, they are separated by deconvolution; otherwise, the sensitivity would be reduced [22]. The peak sharpening method as an easier and simpler alternative to peak deconvolution was used for the first time to separate overlapping peaks.

## 2. Materials and Methods

### 2.1. Phosphor Synthesis

Yttrium (III) oxide (Y_2_O_3_, Alfa Aesar, purity 99.9%), europium (III) oxide (Eu_2_O_3_, Alfa Aesar, purity 99.9%), and molybdenum (VI) oxide (MoO_3_, Alfa Aesar, purity 99.95%) were used as starting materials without further purification. Herein, eight samples with abbreviated names, YMO2Eu, YMO8Eu, YMO10Eu, YMO20Eu, YMO40Eu, YMO50Eu, YMO80Eu, and EuMO, were synthesized by a high-temperature solid-state method (the exact doping concentrations of Eu^3+^ ions and sample formulas are provided in Table 1). In an optimized synthesis, the stoichiometric amounts of Y_2_O_3_, Eu_2_O_3_, and MoO_3_, were homogenized by dry grinding in methanol in an agate mortar and heated for 4 h in open crucibles at 300 °C. To complete the reaction, the products were cooled down to room temperature, finely ground, and annealed at 800 °C in an air atmosphere for 4 h. The collected powders were white, and with an increase in the doping concentration of Eu^3+^ ions, they became pale pink.

### 2.2. Characterization

The crystal structure of the samples was investigated by X-ray diffractometer (XRD) (Cu-Kα1,2 radiation, λ = 0.1540 nm, Rigaku SmartLab, Tokyo, Japan) at room temperature. The measurements were recorded over the 10°–70° range, with 0.02° step size and 10°/min counting time. Built-in PDXL2 v2.1 package software was used to obtain relevant parameters of the structural analysis. The morphology of the prepared sample was defined by a field emission gun MIRA3 (TESCAN, Brno Kohoutovice, Czech Republic) field emission scanning electron microscope (FE-SEM), the samples were coated with a thin layer of Au using a typical sputtering technique (Polaron SC502-Fison Instruments, Glasgow, UK). The samples’ UV–VIS diffuse reflection spectra (DRS) were recorded with a UV-3600 UV-VIS-NIR spectrophotometer (Shimadzu, Kyoto, Japan) in the visible spectral region and BaSO_4_ was used as the reflectance standard. Emission spectra were measured using the Ocean Optics (Orlando, FL, USA) FX UV-VIS Spectrometer with the excitation light source–405 nm Ocean Optics (Orlando, FL, USA) Insight LED module working in continuous mode and controlled by LDC-1. The temperature-dependent emission spectra were recorded over 300 K to 650 K with a 25 K step. The temperature of the samples was controlled by using a MicroOptik MHCS400 (Breskens, Netherlands) liquid nitrogen-cooled heating-cooling stage. Lifetime measurements were carried out at room temperature using the Rohde & Schwarz RTC1002 (Munich, Germany) two-channel oscilloscope (1 µs temporal resolution) paired with the Hamamatsu (Shizuoka, Japan) H10722-20 photomultiplier tube and by exciting the samples with a square wave modulated 405 nm Ocean Optics Insight fiber-coupled LED (LSM 405A, Orlando, FL, USA) controlled by Ocean Insight LDC-1 Single Channel LED Controller.

## 3. Results and Discussion

### 3.1. Structural and Morphological Properties

**Figure 1:**
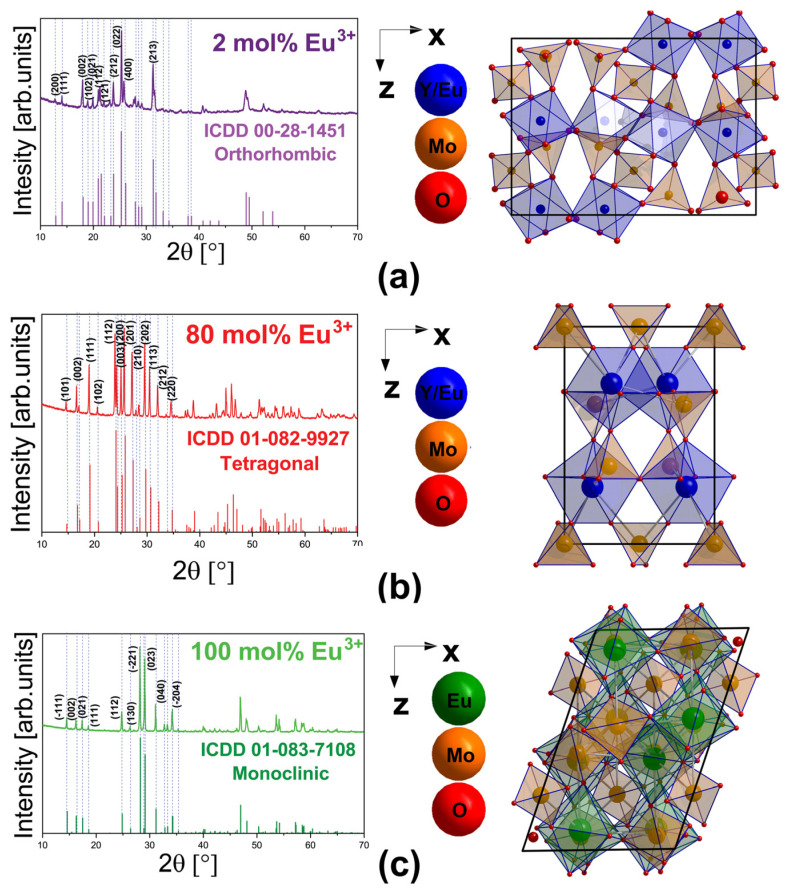
XRD patterns and schematic presentation of crystal structure in (**a**) orthorhombic YMO2Eu with corresponding ICDD Card No. 00-028-1451; (**b**) tetragonal YMO80Eu with corresponding ICDD Card No. 01-082-9927; and (**c**) monoclinic EuMO with corresponding ICDD Card No. 01-083-7108. Schematic presentations of crystal structures were built in Diamond Crystal and Molecular Structure Visualization software v5.0.

The effect of the Eu^3+^ ions doping concentration on the crystal structure of the synthesized Y_2−x_Eu_x_Mo_3_O_12_ phosphors was analyzed by X-ray diffraction measurements. In the samples with smaller Eu^3+^ doping concentrations (YMO2Eu and YMO8Eu) diffraction patterns correspond to the orthorhombic crystal structure with Pbcn (60) space group composed of corner-shared (MoO_4_) tetrahedrons and (YO_6_) octahedrons (Figure 1a and Figure S1a). Further increase of the Eu^3+^ ions concentration (YMO10Eu and YMO20Eu) leads to a mixture of two phases: orthorhombic and tetragonal as shown in the Supporting Information file (Figure S1b). An additional increase of Eu^3+^ ions concentration leads to a single-phase tetragonal crystal structure with space group P-421m (113) in YMO40Eu, YMO50Eu, and YMO80Eu samples (Figure 1b and Figure S1c). Y_2_Mo_3_O_12_ comprises corner-shared (MoO_4_) tetrahedrons and (YO_7_) heptahedrons in the tetragonal structure. Complete substitution of Y^3+^ with Eu^3+^ ions results in the formation of pure Eu_2_Mo_3_O_12_ whose crystal structure is monoclinic with space group C_2_/C (15) (Figure 1c) and is formed of (MoO_4_) tetrahedrons/(MoO_5_) pentahedrons and (EuO_8_) polyhedrons linked by corners.

Unique ranges of ionic radii of trivalent rare earth dictate the fact that the RE_2_M_3_O_12_ family adopts several crystal structures, resulting in different preferences for coordination environments. Smaller RE ions such as Er, Tm, Yb, and Lu usually form 6-coordinate structures, while the case of larger RE ions (such as La, Eu, Gd, Ho, Sm, and Tb) is that they generally have high coordination numbers (7 or 8) [8]. In addition, yttrium ion can be distinguished as a pseudo-lanthanide, as it has very similar properties to lanthanides. Y^3+^ ionic radius is borderline between the smaller lanthanides forming 6-coordinated structures, and those preferring structures with higher coordination numbers [8]. Therefore, the replacement of Y^3+^ with larger size Eu^3+^ ions results in the transformation from orthorhombic (for low doping concentrations) through tetragonal (for high doping concentrations) reaching monoclinic structure for full replacement in Eu_2_Mo_3_O_12_. 

Crystallite size, as well as the unit cell parameters, and micro strains of the obtained materials, were calculated using the built-in program package PDXL2 (Table 2).

**Figure 2:**
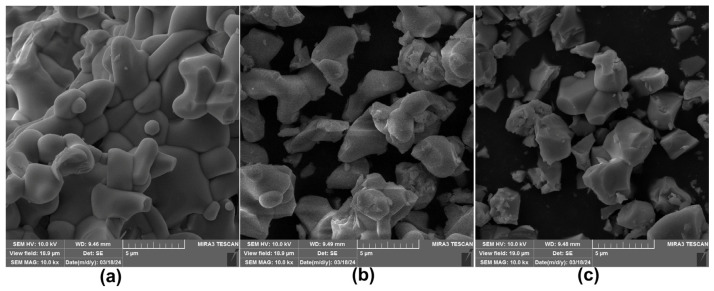
Representative FE-SEM images under 10.0 kx magnification of (**a**) orthorhombic YMO2Eu (**b**) tetragonal YMO80Eu, and (**c**) monoclinic EuMO samples.

FE-SEM images of the representative orthorhombic, tetragonal, and monoclinic Eu³⁺-doped Y_2_Mo_3_O_12_ samples reveal agglomerated particles with irregular, elongated, quasi-spherical shape, as illustrated in Figure 2. A detailed examination of these images indicates that, despite the distinct crystal structures and varying concentrations of Eu^3^⁺ ions, the overall morphology and shape of the particles exhibit notable similarities across all three crystal phases. This uniformity suggests that the Eu^3^⁺ ion doping does not significantly alter the fundamental particle morphology. Furthermore, the particles appear to aggregate into clusters, with the size and distribution of these clusters remaining consistent regardless of the crystal phase or dopant concentration. This observation may imply a common growth mechanism or interaction between the particles during the synthesis process, highlighting the robustness of the material’s structural integrity across different doping levels and crystalline forms.

### 3.2. UV-VIS and Photoluminescent Properties

**Figure 3:**
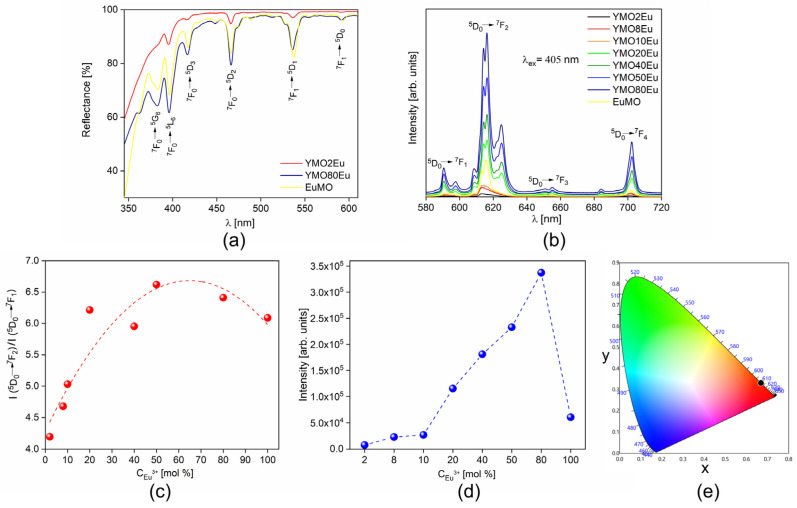
(**a**) Room temperature diffuse reflectance spectra of representative orthorhombic YMO2Eu, tetragonal YMO80Eu, and monoclinic EuMO samples; (**b**) room temperature photoluminescent emission spectra of all Eu^3+^-doped Y_2_Mo_3_O_12_ samples (λex = 405 nm); (**c**) asymmetry ratio as a function of Eu^3+^-dopant ion concentration; (**d**) integrated emission intensity of all Eu^3+^-doped Y_2_Mo_3_O_12_ samples as a function of Eu^3+^ concentrations. The integrated emission intensity was calculated for the whole emission area, in the 580 to 720 nm range for each sample; (e) CIE chromaticity diagram.

Figure 3a presents the diffuse reflectance spectra of representative YMO2Eu, YMO80Eu, and EuMO samples in the 345–610 nm spectral range where typical transitions originating from Eu^3+^ ions can be observed. The absorption peaks of Eu^3+^ ions, which are located at 381, 396, 416, 466, 537, and 593 nm correspond to the following electronic transitions ^7^F_0_ → ^5^G_6_, ^7^F_0_ → ^5^L_6_, ^7^F_0_ → ^5^D_3_, ^7^F_0_ → ^5^D_2_, ^7^F_1_ → ^5^D_1_, and ^7^F_1 _→ ^5^D_0_, respectively, with the highest absorption placed at ~396 nm [23,24]. 

**Figure 4:**
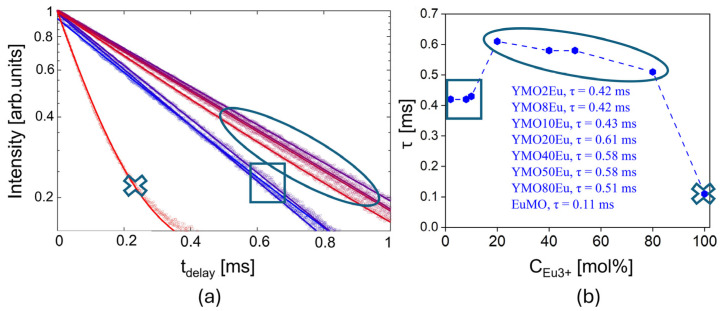
(**a**) Recorded lifetime decay curves for samples YMO2Eu-YMO10Eu with orthorhombic structure (square shape), YMO20Eu-YMO80Eu samples (round shape), and monoclinic EuMO (x shape) and (**b**) calculated luminescence decay times as a function of dopant Eu^3+^ concentration.

The room temperature photoluminescent emission spectra of the synthesized samples recorded upon 405 nm excitation are presented in Figure 4b. All materials show typical Eu^3+^ red emission, with the most intense peak placed at ~616 nm corresponding to ^5^D_0_→^7^F_2_ electric-dipole transition. Also, three others characteristic Eu^3+^ ion transitions can be noticed: magnetic dipole ^5^D_0_→^7^F_1_ placed at ~590 nm, as well as ^5^D_0_→^7^F_3_ placed at ~655 nm, and ^5^D_0_→^7^F_4_ transition placed at ~702 nm.

^5^D_0_→^7^F_1_ is a magnetic-dipole transition and does not depend on a local environment. However, the ^5^D_0_→^7^F_2_ electric-dipole transition is a hyper-sensitive one, and it is very dependent on the minor changes in the local environment of the Eu^3+^ ions. Therefore, theoretically, the ratio of the integrated intensity of the ^5^D_0_→^7^F_2_ and ^5^D_0_→^7^F_1_ transitions, known as the asymmetry ratio, can be considered indicative of the reduction of symmetry of the coordination environment around the Eu^3+^ ion and is given by Equation (1):

(1) R=I(D05→F27)I(D05→F17)

Figure 3c presents the asymmetry ratio as a function of Eu^3+^-dopant ion concentration. The ratio increases in the set of samples with orthorhombic structure (from YMO2Eu to YMO10Eu), followed by a further increase of the ratio in the samples with the tetragonal structure which among them have similar values (from YMO20Eu to YMO80Eu) The asymmetry ratio value for monoclinic EuMO is relatively similar to the samples with the tetragonal structure. The high value of the asymmetry ratio as a function of dopant ion concentration indicates low symmetry of the Eu^3+^ surroundings in the molybdate crystal. 

Figure 3d shows integrated emission intensity as a function of Eu^3+^ dopant concentration. The emission intensity increases slightly in the orthorhombic structure for 2 mol% Eu^3+^ and 8 mol% Eu^3+^ concentrations. With a further increase in the Eu^3+^ ions concentration, there is a significant rise in the emission intensity having the highest value for the tetragonal YMO80Eu sample showing that the YMO host can be heavily doped without a decrease in the emission intensity. Complete substitution of the Y^3+^ ions with Eu^3+^ in the case of monoclinic EuMO leads to a clear decrease in the emission intensity. 

CIE chromaticity coordinates (x, y) were derived from the photoluminescent spectra and shown in Figure 3e. For all the samples, CIE coordinates are practically identical (x = 0.669, y = 0.331) and placed in the red-orange fragment of the diagram. Color purity of 99% (dominant wavelength–611 nm) was also calculated using the Osram color calculator.

Figure 4a displays the photoluminescent lifetime decay curves of all samples recorded at room temperature under 405 nm excitation. To obtain the value of the lifetime (τ), acquired data were fitted to a simple single exponential function:

(2) $$I\left(t\right)=I_{0}e^{-\frac{t}{\tau }}+\mathrm{noise}$$

where *I(t)* is the corresponding PL intensity at time t, *I*_0_ is the initial PL intensity, and *τ* is the photoluminescent lifetime; corresponding fitting parameters as well as goodness of fit values are presented in Table S1 (See Supporting Information). Figure 4b represents calculated lifetime values for all the Y_2−x_Eu_x_Mo_3_O_12_ samples, with the longest decay time observed in the case of the YMO20Eu sample showing τ = 0.61 ms value. Obtained decay times follow a similar trend as emission intensity. Values are lower in the orthorhombic structure for 2mol% Eu^3+^ and 8mol% Eu^3+^ concentration, reaching slightly higher values for tetragonal structure and showing a strong decrease for monoclinic Eu_2_Mo_3_O_12_.

The observed lifetime of an excited state is influenced by both radiative and nonradiative processes. The radiative rate *A_R_* represents the rate at which the excited state decays by emitting a photon. Nonradiative rates *A_NR_* represent all other decay pathways, such as vibrational relaxation, that do not involve photon emission. The observed lifetime is the reciprocal of the total decay rate, which is the sum of radiative and nonradiative rates:

(3) $$\frac{1}{\tau }=A_{R}+A_{NR}$$

A longer observed lifetime indicates a higher radiative rate relative to the nonradiative rate, while a shorter lifetime suggests more significant nonradiative processes. Thus, the relationship between these rates determines how efficiently luminescence occurs. The phase of the host material affects both radiative and non-radiative rates by influencing the interaction of the luminescent centers with their environment, impacting how efficiently energy is radiated or lost to non-radiative processes. At higher concentrations, cross-relaxation between activator ions becomes significant, further increasing non-radiative rate.

To determine the temperature stability of emission and the application potential of these materials in LEDs, temperature-dependent photoluminescence measurements were recorded for the YMO80Eu sample. Emission intensity shows high-temperature stability in the maximal operating temperature of LEDs which is, depending on the manufacturer, usually considered to be 100 °C [25,26]. For the YMO80Eu sample, the emission intensity at 100 °C remains at 98% of its initial value at room temperature.

### 3.3. Temperature-Dependent Photoluminescent Properties

**Figure 5:**
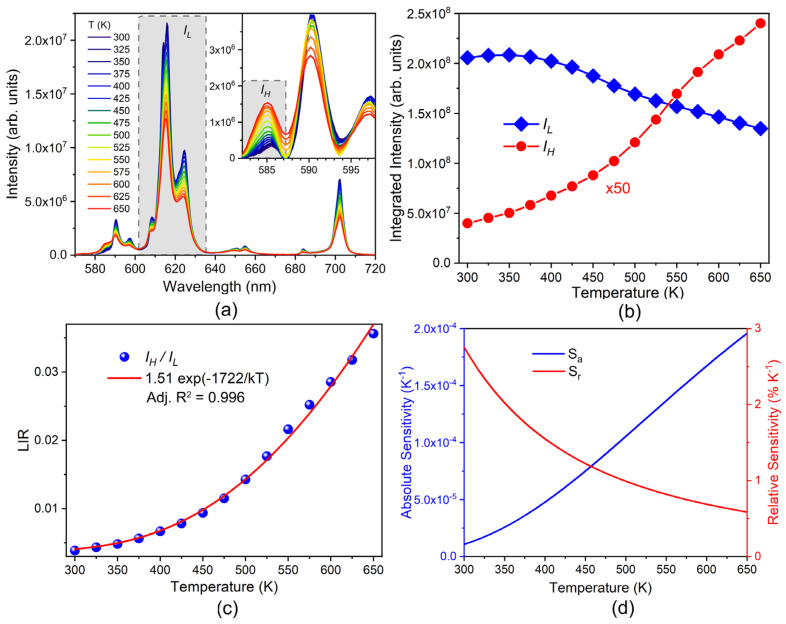
(**a**) Temperature-dependent YMO80Eu emission spectra. (Inset) separation of ^5^D_1_→^7^F_3_ transition by peak sharpening method. (**b**) Integrated intensities of ^5^D_0_→^7^F_2_ and ^5^D_1_→^7^F_3_ emissions, (**c**) their LIR and fit to the Boltzmann relation, and (**d**) corresponding absolute and relative sensitivities. LumThools performs the fits [41].

Temperature-dependent emission spectra of the sample with the highest emission intensity (YMO80Eu) are presented in Figure 5a. The emission intensities of ^5^D_0_→^7^F_1,2,3,4_ peaks slowly decrease with increasing temperature. However, at 585 nm [27], the peak originating from ^5^D_1_→^7^F_3_ emission increases with temperature due to the thermalization from the ^5^D_0_ level. As its position overlaps with the ^5^D_0_→^7^F_1_ transition and because of the thermal peak broadening, this peak is not distinctly resolved. Using spectral areas separated by wavelength in luminescence thermometry here would result in lowered sensitivity as there are contributions from other transitions with different temperature trends. In our previous paper, we demonstrated how peak deconvolution can be used for separating transitions of different origins and the staggering effect it has on the increase of sensitivities [22]. However, peak deconvolutions are difficult to properly conduct as they require multiple-peak fitting at every measured temperature. Here we propose an alternative in separating overlapping peaks (here ^5^D_0_→^7^F_1_ and ^5^D_1_→^7^F_3_) by the method called numerical resolution enhancement or peak sharpening [28,29], where the intensity is modified by:

(4) $$i_{\mathrm{sharp}}\left(\lambda \right)=i\left(\lambda \right)-w_{1}\frac{d^{2}i\left(\lambda \right)}{d\lambda^{2}}+w_{2}\frac{d^{4}i\left(\lambda \right)}{d\lambda^{4}}$$

where *w_1_* and *w_2_* are the weighting factors. The result of the peak sharpening is given in Figure 5a inset, where the ^5^D_1_ emission is distinctly separated from the ^5^D_0_→^7^F_1_. Note that the peak sharpening method does not affect the peak positions or the integrated area. 

The trends of integrated intensities of ^5^D_0_→^7^F_2_ (from Figure 5a) and ^5^D_1_→^7^F_3_ (from Figure 5a inset) with increasing temperature are given in Figure 5b. The energetically lower ^5^D_0_ emission (L) slowly decreases, while the energetically higher ^5^D_1_ (H) emission increases with temperature to the expense of the former level population. The luminescence intensity ratio (LIR) of those two emissions is given by the Boltzmann distribution [30]:

(5) $$LIR=\frac{I_{H}}{I_{L}}=B\exp\left(-\frac{\Delta E}{kT}\right)$$

where *B* is the temperature invariant parameter, *k* = 0.695 cm^−1^ K^−1^ is the Boltzmann constant, *T* is the temperature in Kelvins, and Δ*E* is the energy separation between thermalized levels. The temperature invariant *B* parameter is related to the ratio of radiative transition probabilities from the ^5^D_1,0_ levels, respectively:

(6) B∝A(D15→F37)A(D05→F27)

The fit of Equation (5) to the ratios of integrated intensities in Figure 5b is presented in Figure 5c. Good fit quality proves the effectiveness of the thermalization from the ^5^D_0_ to the ^5^D_1_ level even at room temperature. The fitted Δ*E* corresponds to the energy difference of the ^5^D_0_ and ^5^D_1_ levels [31].

The relative and absolute sensitivity are calculated by, respectively:

(7) $$S_{a}=LIR\frac{\Delta E}{kT^{2}},\;S_{r}=\frac{\Delta E}{kT^{2}}\cdot 100\%$$

and the results are given in Figure 5d. At room temperature, the relative sensitivity has a relatively high value of 2.8% K^−1^ compared to other single lanthanide-doped probes. Due to the high energy gap this Eu^3+^-doped sensor probe is best employed for higher temperatures [32]. The comparison of sensitivities with other Eu^3+^-doped hosts at elevated temperatures, by the LIR method, is given in Table 3. The LIR using peak sharpening is comparable to the LIR by ^5^D_1_→^7^F_1_ and ^5^D_0_ emissions, as conventionally performed [33].

**Table 3:** Hosts for Eu^3+^-doped luminescence thermometry probes, measured temperature ranges by the LIR method, and absolute and relative sensitivities at 500 K.

Host	Range (K)	S_a_ (K^−1^) @500 K	S_r_ (% K^−1^) @500 K	Ref.
YVO_4_	300–750	0.0007	0.6	[34]
Ca_7_V_4_O_17_	333–773	0.01	0.98	[35]
ZnO	100–500	0.02	1.2	[36]
LiNbO_3_	300–750	0.14	1.18	[37]
YAG	300–850	0.033	1.01	[38]
TiO_2_	293–533	0.164	1.02	[39]
SrZrO_3_	300–550	0.005	0.8	[40]
NaEuF_4_	298–523	/	0.97	[33]
Y_2_Mo_3_O_12_	300–650	0.015	0.99	This work

## 4. Conclusions

This study discussed changes in the crystal structure and photoluminescent properties of Y_2_Mo_3_O_12_ caused by Eu^3+^ doping. The findings can be summarized as:

- Incorporation of Eu^3+^ ions affects the crystal structure of Y_2_Mo_3_O_12_; the existence of an orthorhombic via tetragonal to monoclinic phase transition, depending on the concentration of dopant Eu^3+^ ions is shown.
- The luminescence intensity of Eu^3+^-doped Y_2_Mo_3_O_12_ samples was enhanced by increasing the Eu^3+^ dopant concentration up to optimum 80 mol% of Eu^3+^.
- Emission intensity shows high-temperature stability in the maximal operating temperature of LEDs and the YMO80Eu sample remains at 98% of its initial value at 100 °C.
- An optimized 80 mol% Eu^3+^-doped Y_2_Mo_3_O_12_ sample is proposed as a promising thermal probe with a relative sensitivity of 2.8% K^−1^ at room temperature.
- The peak sharpening method is equally effective as the peak deconvolution method in the separation of overlapping peaks for the luminescence intensity ratio method.

To summarize, the red-emitting Eu^3+^-doped Y_2_Mo_3_O_12_ system can be heavily doped, showing high-temperature emission stability and a high value of relative sensitivity. Therefore, it shows potential in both lightning and luminescent thermometry fields.

## Figures and Tables

**Table 1:** Chemical formulas of the synthesized Y_2−x_Eu_x_Mo_3_O_12_ samples.

Eu^3+^ Concentration (mol %)	Sample Formula Y_2−x_Eu_x_Mo_3_O_12_	Abbreviated Sample Name
2	Y_1.96_Eu_0.04_Mo_3_O_12_	YMO2Eu
8	Y_1.84_Eu_0.16_Mo_3_O_12_	YMO8Eu
10	Y_1.8_Eu_0.2_Mo_3_O_12_	YMO10Eu
20	Y_1.6_Eu_0.4_Mo_3_O_12_	YMO20Eu
40	Y_1.2_Eu_0.8_Mo_3_O_12_	YMO40Eu
50	Y_1_Eu_1_Mo_3_O_12_	YMO50Eu
80	Y_0.4_Eu_1.6_Mo_3_O_12_	YMO80Eu
100	Eu_2_Mo_3_O_12_	EuMO

**Table 2:** Calculated structural parameters for the synthesized single-phase Y_2−x_Mo_3_O_12_:xEu^3+^ (x = 0.04; 0.16; 0.8; 1; 1.6, 2) phosphors.

Sample	YMO2Eu	YMO8Eu	YMO40Eu	YMO50Eu	YMO80Eu	EuMO
	orthorhombic	orthorhombic	tetragonal	tetragonal	tetragonal	monoclinic
No.	01-075-5430	01-075-5430	01-082-9927	01-082-9927	01-082-9927	01-083-7108
R_wp_ (%)	12.43	10.21	6.14	7.35	5.67	7.85
R_p_ (%)	8.16	6.77	4.62	5.29	4.29	5.06
R_e_ (%)	3.17	3.23	3.04	3.26	3.35	2.78
GOF	3.9263	3.1638	2.0181	2.2523	1.6941	2.8204
CS (Å)	165(5)	288(50)	455(12)	366(9)	299(8)	418(9)
Strain	0.13(2)	0.83(19)	0.0660	0.12(17)	0.13(6)	0.11(5)
a	13.755(2)	13.7340(14)	7.3230(4)	7.3357(2)	7.3625(9)	7.591(3)
b	9.909(3)	9.9237(14)	7.3230(4)	7.3357(2)	7.3625(9)	11.4651(5)
c	9.906(2)	9.9370(11)	10.6292(6)	10.6551(4)	10.6979(9)	11.5021(5)

CS—crystallite size; Rwp—the weighted profile factor; Rp—the profile factor; Re—the expected weighted profile factor; GOF—the goodness of fit.

## Data Availability

The raw data supporting the conclusions of this article will be made available by the authors upon request.

## Supplementary Materials

The following supporting information can be downloaded at: https://www.mdpi.com/article/10.3390/ma17174267/s1, Figure S1: XRD images of (a) orthorhombic YMO8Eu (b) mixed tetragonal and orthorhombic YMO10Eu, YMO20Eu, and (c) tetragonal YMO40Eu, YMO50Eu phosphors; Table S1: S1 Emission decay lifetimes parameters for single exponential fitting at peak emission wavelengths under 405 nm excitation and GOF values.
