# Supplementary material for: Structure–Dopant Concentration Relations in Europium-Doped Yttrium Molybdate and Peak-Sharpening for Luminescence Temperature Sensing
Source: Materials (Basel). 2024 Aug 28;17(17):4267. doi: 10.3390/ma17174267 (PMC11396498; doi:10.3390/ma17174267)
Supplement: Supplementary file 1 [file materials-17-04267-s001.zip › materials-3144729-Supplementary Materials.pdf]

Structure–dopant concentration relations in Europium -doped Yttrium Molybdate and peak-sharpening for luminescence temperature sensing

*Tamara Gavrilović *, Aleksandar Ćirić*, Mina Medić, Zoran Ristić, Jovana Periša, Željka Antić, Miroslav Dramićanin*

Center of Excellence for Photoconversion, Vinča Institute of Nuclear Sciences – National Institute of the Republic of Serbia, University of Belgrade, P.O. Box 522, Belgrade 11001, Serbia

**Corresponding author email: tashichica@gmail.com, aleksandar.ciric@ff.bg.ac.rs*

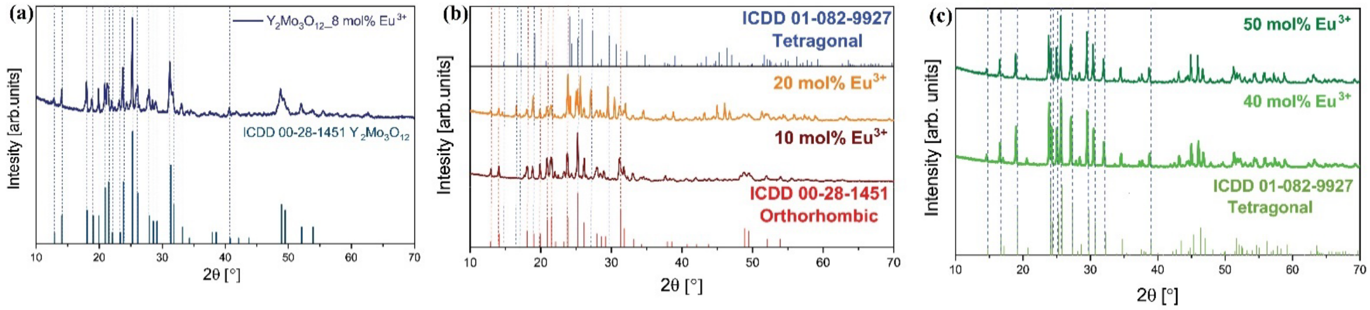

Figure S1 XRD images of a) orthorhombic YMO8Eu b) mixed tetragonal and orthorhombic YMO10Eu, YMO20Eu, and c) tetragonal YMO40Eu, YMO50Eu phosphors.

Table S1 Emission decay lifetimes parameters for single exponential fitting at peak emission wavelengths under 405 nm excitation and GOF values.

Sample	n (mol% Eu^{3+})	τ (ms)	I_0	Noise	GOF (R^2)
**YMO2Eu**	2	0.42	0.91763	0.87581	0.9989
**YMO8Eu**	8	0.42	0.97613	$5.4761e^{-13}$	0.9995
**YMO10Eu**	10	0.43	0.99071	$2.3012e^{-12}$	0.9993
**YMO20Eu**	20	0.61	1.00400	$1.64e^{-13}$	0.9993
**YMO40Eu**	40	0.58	1.00310	$4.4075e^{-13}$	0.9998
**YMO50Eu**	50	0.58	0.98919	0.0013669	0.9998
**YMO80Eu**	80	0.51	0.96629	0.027259	0.9998
**EuMO**	100	0.11	0.87581	0.11396	0.9976
